# Recommended or high daily intakes of plant stanol esters do not affect *ex vivo* T-cell derived cytokine production in immunologically healthy volunteers

**DOI:** 10.1017/S0007114524001363

**Published:** 2024-10-28

**Authors:** Lieve van Brakel, Florence Brüll, Anissa Lasfar, Willem Zwaan, Arienne de Jong, Ronald P. Mensink, Jogchum Plat

**Affiliations:** Department of Nutrition and Movement Sciences, Institute of Nutrition and Translational Research in Metabolism (NUTRIM), Maastricht University, Maastricht, Netherlands

**Keywords:** Plant stanols, Immune system, Cytokine production, Healthy volunteers

## Abstract

A well-functioning immune system requires balanced immune responses. *In vitro* studies have shown that plant stanols contribute to restoring the T-helper (Th)1/Th2 ratio when it is imbalanced. However, effects of plant stanols on healthy immune responses are unknown. Therefore, we studied effects of recommended (2·5 g/d) or high (9·0 g/d) plant stanol intakes on the Th1/Th2 cytokine balance in immunologically healthy subjects. In two RCTs, peripheral blood mononuclear cells (PBMCs) were isolated, cultured, and stimulated with 5 µg/ml Phytohemagglutinin-M to study *ex vivo* cytokine production. In the first study, twenty participants consumed margarines (2·5 g/d plant stanols) or control for three weeks. In the second study, nineteen participants consumed margarines and yogurts (9·0 g/d plant stanols) or control for four weeks. T-cell cytokine concentrations were measured in culture medium and in study 2 a standardized Th1/Th2 index was calculated. Serum lipids and non-cholesterol sterols were also measured. Compliance was confirmed by significant increases in serum total cholesterol (TC)-standardized sitostanol and campestanol levels in both studies. Changes in *ex vivo* cytokine production and Th1/Th2 index did not differ between intervention and control groups. In the first study, no statistically significant changes were observed in lipid and lipoprotein concentrations. In the second study, LDL cholesterol significantly decreased compared to control (–0·77 (–1·11, –0·42) mmol/l; *P* < 0·001). Recommended (2·5 g/d) or high (9·0 g/d) intakes of plant stanols did not alter PBMC *ex vivo* cytokine production in immunologically healthy subjects. This suggests that plant stanols might only affect immune function when Th1/Th2 immune responses are imbalanced.

The human immune system can initiate a wide variety of cellular and humoral responses to infectious challenges, in which numerous cells, cytokines and other signalling molecules work together in a well-regulated manner. If the delicate balance between these responses is somehow disturbed, it may underlie the occurrence of various immune-related diseases^(1)^. For example, a dominant response of T-helper 1 (Th1) cells plays an important role in inflammatory diseases and autoimmune diseases, whereas a shift towards a Th2 dominant immune response has been causally linked to asthma and allergic reactions^(1–3)^.

Previous studies have suggested that plant stanol esters modify Th cell responses. Plant stanols are the saturated derivatives of plant sterols and are structurally comparable to cholesterol. This characteristic causes plant stanols to lower intestinal cholesterol absorption, e.g. by competing with cholesterol for incorporation into mixed micelles^(4–6)^. However, plant stanols may also have immunomodulatory effects. First, we have shown that plant sterols and stanols increased the activity of Th1 cells *in vitro.* Increased Th1 activity was reflected by increased production of the cytokines interferon (IFN)*γ* and interleukin (IL)-2, while the number and activity of regulatory T (Treg) cells were simultaneously increased^(7)^. Next, plant stanol induced effects on *in vitro* T-cell behaviour in cells isolated from asthma patients were evaluated, as these patients are characterized by Th2 dominant immune responses (i.e. increased IL-13 and immunoglobulin (Ig)E production in response to allergic stimuli)^(8)^. In murine models, this undesirable skewing can be reversed by the stimulation of Th1 and Treg cells^(9,10)^. As expected, plant stanols shifted the *in vitro* T-helper cell response away from the Th2 dominant profile in cells of asthma patients, but no apparent changes were detected in cells of healthy controls^(8)^. In a follow-up randomized placebo-controlled trial, we have shown that plant stanol ester consumption indeed stimulated the Th1-mediated vaccination response and simultaneously lowered the Th2 response in asthma patients^(11)^. Finally, we showed that plant stanols improved vaccine-specific antibody responses and cytokine production in subjects with overweight or obesity after a COVID-19 vaccination^(12)^. To the best of our knowledge, it is unknown whether plant stanol ester consumption also affects immune cell behaviour and consequent immune responses in an immunologically healthy population *in vivo*. In other words, is it possible that plant stanol ester consumption disturbs balanced immune responses in healthy subjects, ultimately leading to negative side effects caused by overstimulation of e.g. Th1 cells? Therefore, the aim of the present study was to examine whether plant stanol ester consumption at recommended (2·5 g/d) or even at high (9·0 g/d) intakes affects the Th1/Th2 cytokine balance in immunologically healthy subjects.

## Methods

### Design

Study 1 evaluated the effects of a recommended dose of plant stanols (2·5 g/d^(13)^) on lipid metabolism in a population characterized by elevated fasting serum triglycerides^(14)^. In total, 28 participants completed this study. All participants started with a one-week run-in period using a control margarine without added plant stanol ester. Then, fourteen participants received a plant stanol ester enriched margarine for three weeks, resulting in a plant stanol consumption of 2·5 g/d, while the other fourteen participants continued using the control margarines. The main objective of study 2 was to examine if there was a dose-dependent relation between plant stanol ester consumption (3·0, 6·0, or 9·0 g/d) with changes in serum LDL cholesterol concentrations and fat-soluble anti-oxidants^(15)^. In total, 93 participants completed this study. All participants started with a three-week run-in period, during which they consumed control margarines and yoghurts without plant stanol esters. Thereafter, 22 participants continued to consume the control products, while the other 71 participants received plant stanol ester enriched margarines and yoghurts for four weeks, resulting in a plant stanol intake of 3·0 g/d (*n* 24), 6·0 g/d (*n* 22), or 9·0 g/d (*n* 25). Intervention periods of both studies were long enough to observe possible changes in cytokine production in the plant stanol ester groups^(12)^. For this current study, only participants from the control group and the 9·0 g/d group were included; we reasoned that effects on cytokine production would be most pronounced in the group with the highest intake, if any were present. In both studies, peripheral blood mononuclear cells (PBMCs) were isolated and cultured from a whole blood sample to evaluate potential effects on cytokine production. Participants in the intervention groups were selected for PBMC isolation based on the following criteria: the absence of self-reported asthma, allergies, eczema, or inflammatory diseases; sex to reach an equal number of men and women; BMI < 27 kg/m^2^; aged between 18 and 65 years; non-smokers. Participants in the control groups were matched to those in the intervention groups based on age, sex, and BMI, and had to fulfill the same inclusion and exclusion criteria. This procedure resulted in the selection of subgroups from the original two studies: twenty otherwise healthy participants with hypertriglyceridemia from study 1 and nineteen healthy volunteers with slightly elevated serum total cholesterol (TC) concentrations (5·0–8·0 mmol/l) from study 2.

In both studies, the plant stanol ester blend mainly contained sitostanol (79 %) and campestanol esters (16 %). Detailed information about the design and the participants of both studies have been published earlier^(14,15)^. This study was conducted according to the guidelines laid down in the Declaration of Helsinki and all procedures involving human subjects were approved by the ethics committee of Maastricht University (MEC 07-3-023 and MEC 07-3-059, respectively). Written informed consent was obtained from all subjects.

### Cell culture

In both studies, blood was sampled for PBMC isolation at the end of the run-in period and at the end of the intervention period based on a previously described method^(8)^. From the subjects in study 1, PBMCs were freshly isolated from heparinized blood using Lymphoprep (Nycomed Pharma, Asker, Norway) gradient centrifugation according to the manufacturer’s instructions. Cells were seeded (10^6^/ml) in a 24-wells flat-bottom culture plate (Corning Incorporated, Corning, New York, USA) and cultured in RPMI 1640 medium (Gibco, Thermo Fisher Scientific, Waltham, Massachusetts, USA) supplemented with 1 % sodium pyruvate, 1 % penicillin/streptomycin and 1 % human serum pool, heat inactivated at 56°C for 30 min. 5 µg/ml Phytohemagglutinin-M (Roche Diagnostic Systems, Mannheim, Germany) was used to induce T-cell proliferation. After 52 h, the supernatant was removed, centrifuged, and stored at –80°C until further analysis.

PBMCs from the subjects in study 2 were isolated and cultured as mentioned above. However, in these experiments the culture medium was supplemented with 80 % autologue serum, heat inactivated at 56°C for 30 min, to mimic the elevated plant stanol serum concentrations of the participants during the test period of 9·0 grams per day.

### Lipid, lipoprotein and non-cholesterol sterol concentrations

In both studies, blood was sampled in 10 ml tubes (study 1: one 10 ml serum tube and 1 EDTA tube; study 2: one 10 ml serum tube; Becton–Dickinson Vacutainer Systems, Breda, The Netherlands) at the end of the run-in period and at the end of the intervention period. Serum tubes clotted at room temperature for at least one hour and were centrifuged at 2000 × *g* for 30 min at 4°C. Plasma tubes were centrifuged immediately at 2000 × *g* for 30 min at 4°C. Serum and plasma aliquots were stored at −80°C until further analysis. Plasma (study 1) or serum (study 2) concentrations of non-cholesterol sterols, i.e. plant sterols, plant stanols, and lathosterol, were measured using GC-MS as described earlier^(15)^. In both studies, serum concentrations of TC (Roche Diagnostic Systems, Mannheim, Germany), HDL cholesterol (precipitation method; Roche Diagnostic Systems, Mannheim, Germany) and triglycerides with correction for free glycerol (GPO Trinder; Sigma-Aldrich, Saint Louis, Missouri, USA) were analyzed enzymatically in samples collected at the end of the run-in period and at the end of the intervention. Serum LDL cholesterol concentrations were calculated using the Friedewald formula^(16)^.

### Cytokine production

Sandwich ELISAs were used for the quantification of the Th1 cytokine IFN*γ* (Perbio Science International Netherlands B.V., Breda, The Netherlands) and the Th2 cytokine IL-4 (eBioscience, Thermo Fisher Scientific, Waltham, Massachusetts, USA) in the culture medium from the participants in study 1. In study 2, cytokine concentrations were measured in the culture medium using a custom made electrofluorescence cytokine multiplex assay for analysis of the cytokines IL-2, IL-12, IFN*γ*, IL-4, IL-13, IL-10, and IL-17 (Meso Scale Discovery, Rockville, Maryland, USA). A Th1/Th2 index was calculated with standardized values of (IFN*γ* + IL-2)/(IL-4 + IL-13) to adjust for differences in degrees of expression between the four cytokines. This was done by setting the group average concentration of each cytokine at each time point to 100 and by calculating the relative difference to this average for each sample. With these standardized values, the Th1/Th2 index was calculated for each participant and group of study 2, as described earlier^(17)^. Although this index has not formally been validated, it indicates changes in cytokine production in either a Th1 or Th2 direction^(17,18)^. The biochemical analyses to analyze these secondary outcomes were performed shortly after finishing each original study.

### Statistics

All data are presented as mean ± sd or median (IQR) unless indicated otherwise. In samples from both studies, changes in the capacity of PBMCs to produce cytokines were calculated by subtracting concentrations at the end of the run-in period from concentrations at the end of the intervention period. Similar calculations were performed for changes in lipids, lipoproteins, and TC-standardized non-cholesterol sterol levels. Differences between the changes from participants in the intervention and the control groups were compared using an unpaired student’s *t* test or Mann–Whitney U test as appropriate. *P*-values < 0·05 were considered to be statistically significant. All data was analyzed using SPSS 26 for Macintosh (IBM Corp.).

## Results

### Ex vivo cytokine production and Th1/Th2 index

In study 1 (2·5 g/d plant stanols *v*. control), changes in IL-4 and IFN*γ* concentrations in the culture medium did not differ between the plant stanol and control groups (Table 1). In study 2 (9·0 g/d plant stanols *v*. control), no differences were found in changes between the plant stanol and control groups for the *ex vivo* production of IL-2, IL-12, IFN*γ*, IL-4, IL-13, IL-10 or IL-17 (Table 2). In addition, changes in the calculated Th1/Th2 index were not different between both groups in study 2 (mean difference in changes (95 % CI): 0·03 (–0·23, 0·29), *P* = 0·807; Table 2).

**Table 1.** Changes in Th1 cytokine IFN*γ* and Th2 cytokine IL-4 in study 1 (2·5 g/d plant stanols or control) (Mean values and standard deviations; median values and interquartile ranges)

	Plant stanols (*n* 10)						Control (*n* 10)						
	Pre		Post		Change		Pre		Post		Change		
	Mean	sd	Mean	sd	Mean	sd	Mean	sd	Mean	sd	Mean	sd	Between group *P*-value
Th1: IFN*γ* (pg/ml)	5423	3996	5456	4052	32·6	4376	5472	4272	6280	5371	808	6560	0·759
	Median	IQR	Median	IQR	Median	IQR	Median	IQR	Median	IQR	Median	IQR	
Th2: IL-4 (pg/ml)	16·7	9·05–29·9	16·5	5·78–26·7	–1·10	–2·35–1·49	16·4	8·64–25·0	19·3	12·2–31·5	–0·23	–3·98–6·51	0·739

Data presented as mean ± sd or median (IQR).

**Table 2.** Changes in Th1/Th17 and Th2/Treg cytokines in study 2 (9·0 g/d plant stanols or control) (mean values and standard deviations; median values and interquartile ranges)

	Plant stanols (*n* 10)						Control (*n* 9)						
	Pre		Post		Change		Pre		Post		Change		
	Mean	sd	Mean	sd	Mean	sd	Mean	sd	Mean	sd	Mean	sd	Between group *P*-value
Th1/Th17 cytokines (pg/ml)													
IFN*γ*	5951	1601	6487	2196	–220	1224	6183	2966	5963	3114	536	1945	0·385
IL-2	853	652	663	517	–189	495	1133	1161	804	693	–329	841	0·661
IL-12													
Median	13·3		14·5		0·42		12·8		12·4		–3·71		
IQR	10·3–20·2		8·67–25·8		–4·41–8·94		10·2–110·5		9·71–71·2		–43·6–2·77		0·315
IL-17	228	61·7	219	85·6	–9·11	67·8	226	43·8	211	46·8	–14·3	59·2	0·862
Th2/Treg cytokines (pg/ml)													
IL-4													
Median	77·7		77·0		–0·29		76·1		83·8		3·08		
IQR	72·6–91·9		59·6–98·3		–2·05–9·52		64·6–92·1		63·8–97·5		–4·80–11·8		0·661
IL-10	275	87·4	223	88·7	–52·0	70·0	249	56·5	244	74·4	–4·80	61·1	0·125
IL-13	7650	2329	7185	2877	–466	2927	8129	2901	7111	3193	–1018	2343	0·658
Th1/Th2 index													
Th1/Th2	0·96	0·26	0·94	0·27	–0·02	–0·16; 0·12	0·97	0·43	0·92	0·44	–0·05	–0·31; 0·20	0·807

Data presented as mean ± sd or median (IQR).

### Lipid, lipoprotein and non-cholesterol sterol concentrations

In study 1, intake of plant stanols (2·5 g/d) resulted in an apparent decrease of serum TC, which was not statistically significant (mean difference in changes (95 % CI): –0·49 mmol/l (–1·00, 0·02), *P* = 0·060). Additionally, the apparent reduction in serum LDL cholesterol was not significantly different compared to control (Table 3). Although these reductions did not reach statistical significance, effect sizes were as expected^(19,20)^ and these reductions reached statistical significance in the original cohort^(14)^. In study 2, with a daily plant stanol ester intake of 9·0 g, the statistically significant reductions of serum TC and LDL cholesterol were 0·82 mmol/l (–1·19, –0·44, *P* < 0·001) and 0·77 mmol/l (–1·11, –0·42, *P* < 0·001), respectively (Table 3). Serum HDL-cholesterol and triacylglycerol concentrations remained unaffected in both studies.

**Table 3.** Changes in cholesterol and plant stanol serum concentrations per study (mean values and standard deviations; median values and interquartile ranges)

	Study 1					Study 2				
	Plant stanols 2·5 g/d (*n* 10)		Control (*n* 10)			Plant stanols 9·0 g/d (*n* 10)		Control (*n* 9)		
	Mean	sd	Mean	sd	Between groups *P*-value	Mean	sd	Mean	sd	Between groups *P*-value
Lipids and lipoproteins (mmol/l)										
Cholesterol	–0·46	0·41	0·02	0·65	0·060	–0·84	0·31	–0·03	0·46	< 0·001*
LDL-Cholesterol	–0·46	0·63	0·00	0·70	0·137	–0·79	0·26	–0·02	0·44	< 0·001*
HDL-Cholesterol	–0·01	0·08	0·00	0·12	0·901	0·03	0·12	–0·01	0·05	0·444
TAG	0·01	1·03	0·05	0·89	0·927	–0·17	0·21	0·01	0·19	0·066^#^
Non-cholesterol sterols (µmol/mmol × 10^2^ cholesterol)										
Sitosterol	–28·9	30·3	–4·59	7·29	0·033	–83·5	25·0	1·34	37·1	< 0·001*
Campesterol	–33·3	35·5	–3·14	19·1	0·029	–119	32·4	2·72	38·6	< 0·001*
	Median	IQR	Median	IQR		Median	IQR	Median	IQR	
Sitostanol	17·3	12·8–29·8	–0·04	–0·30–0·00	< 0·001	19·8	17·1–26·3	–0·08	–0·25–0·31	< 0·001*
Campestanol	7·95	5·94–13·4	–0·04	–0·16–0·29	< 0·001	9·85	8·52–15·1	0·01	–0·07–0·21	< 0·001*
	Mean	sd	Mean	sd		Mean	sd	Mean	sd	
Lathosterol	17·1	37·3	3·82	53·6	0·528	29·5	26·4	9·94·	24·8	0·115

Data are depicted as mean ± sd or median (IQR). *P<0.05. *^#^P*<0.10.

As expected, both TC-standardized sitostanol and campestanol levels increased significantly in both studies after plant stanol supplementation, confirming compliance. In participants receiving 2·5 g/d plant stanols, sitostanol increased by 17·3 (12·8–29·8) µmol/mmol × 10^2^ cholesterol and campestanol by 7·95 (5·94–13·4) µmol/mmol × 10^2^ cholesterol (both *P* < 0·001), whereas TC-standardized plant sterol concentrations decreased and TC-standardized lathosterol remained unaffected (Table 3). The increases in serum TC-standardized plant stanols were comparable in participants receiving 9·0 grams of plant stanol esters per day: 19·8 (17·1–26·3) µmol/mmol × 10^2^ cholesterol for sitostanol and 9·85 (8·52–15·1) µmol/mmol × 10^2^ cholesterol for campestanol. In these samples, decreases in plant sterol esters were observed as well and TC-standardized lathosterol remained also unaffected (Table 3). More details on the effects on serum lipids have been published earlier for both studies, which slightly differ from those of the current study due to subgroup analyses^(14,15)^.

## Discussion

We here showed that recommended (2·5 g/d) intakes of plant stanols did not affect *ex vivo* production of cytokines IFN-*γ* and IL-4. We also showed that high (9·0 g/d) intakes of plant stanols did not affect *ex vivo* T-cell derived cytokine production and the Th1/Th2 balance. Both studies were carried out in immunologically healthy volunteers. Previous studies in cells, animals, and humans have shown immune modulating, or more specifically Th1 skewing characteristics of plant sterols and stanols^(7–11)^. However, human *in vivo* data regarding immunomodulating effects of plant stanols were only available in certain patient groups characterized by specific (Th2 skewed) immune disorders, like HIV, asthma, or allergies^(11,21,22)^ but not in healthy subjects. Therefore, the effects of consuming foods enriched with plant stanol esters on immune function in healthy populations without disturbed immune conditions remained unknown. *In vitro* data have suggested that PBMCs from healthy volunteers are not influenced by incubation with sitostanol, whereas cells from asthma patients showed a clear Th1 shift in their cytokine production^(8)^. Stimulating the Th1 response has been shown to be an effective therapy in a murine food allergy model, which is characterized by a Th2 dominant immune response^(23)^. However, Th1 cells play an important role in inflammatory responses and are mediators in autoimmune diseases, and overstimulation should therefore be avoided. We here tested the effects of a daily intake up to 9·0 grams of plant stanols on *ex vivo* cytokine production by PBMCs from immunologically healthy volunteers. No changes were found in the production of Th1, Th2, Th17, and Treg-derived cytokines, not even in the group receiving 9·0 grams of plant stanols per day, which is more than three times the daily recommended intake of 2·5–3·0 g^(13)^. This strongly suggests that the healthy immune system will not be skewed into a Th1 dominant response, nor into the Th2 skewed response, by the consumption of plant stanol ester enriched supplements. Additionally, a Th1 shift as indicated by an increased Th1/Th2 index as we have earlier reported in asthma patients^(11)^, did not occur in this immunologically healthy population. However, this Th1 shift in the Th1/Th2 index occurred after asthma patients received a hepatitis A vaccination, which was not given to the participants in the current study. Future studies should explore if the Th1/Th2 index also remains unaffected after receiving a vaccination in immunologically healthy volunteers.

The mechanism behind the immune modulating effects of plant stanol esters is not fully understood. We have previously shown that activation of Toll-like receptor-2 (TLR2) is essential in the plant sterol and stanol dependent Th1 shift. However, on which cells TLR2 was activated remained unknown^(7)^. Also, the increased activity of Treg cells as suggested by *in vitro* experiments may play a role^(8)^. Moreover, a study by De Smet *et al.* ^(24)^ showed decreased numbers of T-helper cells and Treg cells in intestinal biopsies obtained from volunteers who consumed 4 grams of plant stanols per day. Those results do not necessarily indicate a decrease in both Th1 and Th2 numbers, as this distinction was not made. They could also indicate that the role of the intestinal immune cells would be to signal the cells in the circulation on how to respond. However, this exact mechanism and the relation between immune cells and responses in the intestine and circulation needs to be tested in future studies. Moreover, absolute numbers of cells are not a measurement for total cell activity and might therefore not be indicative for the full effects of plant stanols on Th and Treg cell function. Altogether, the mechanisms behind possible immune modulating effects of plant stanols need further investigation.

Although this study only reflects short-term effects in an *ex vivo* setting, it gives an important indication that the consumption of plant stanol ester enriched products do not affect immune function in immunologically healthy people. However, these results need to be confirmed with more volunteers to exclude the possibility of finding false-negative results, and with participants, who have been specifically selected for the absence of any immune-disorders to minimize variation in the immunological results.

A limitation is that the original studies were designed to examine the effects of plant stanol ester consumption on serum lipid concentrations, and not on cytokine production. Therefore, study populations were selected specifically on characteristics related to lipid metabolism rather than immune function. The immune function of the current study population may not have been optimal, because higher BMI, age, and serum lipids are known risk factors for decreased immune functioning^(12,25)^. However, since the current study population had a BMI < 27 kg/m^2^, serum lipid profiles were only slightly elevated, and the age range was wide, we believe that these factors influenced immune function to a limited extent. Moreover, the methods used to measure cytokine concentrations were robust and therefore, we believe that this study was valid to examine the effects of plant stanols ester consumption on cytokine production. Also, we cannot exclude that some subjects might have had mild allergic complaints that they were unaware of, since we relied on self-reporting of any known allergic and inflammatory diseases rather than official medical tests or reports. Another limitation is that only a subset of participants from these two studies was analyzed, resulting in a small sample size. The effect sizes of the lipid data were highly comparable between the full study population and the subset analyzed here. The only difference was that decreases in some lipids and lipoproteins were not statistically significant in these 10 patients receiving 2·5 g/d plant stanols, due to a smaller sample size.

### Conclusion

Daily recommended intakes of plant stanols (2·5 g/d) did not alter *ex vivo* IFN-*γ* or IL-4 production by PBMCs in immunologically healthy subjects. Additionally, high daily intakes of plant stanols (9·0 g/d) did not affect *ex vivo* T-cell derived cytokine production and the Th1/Th2 balance in immunologically healthy volunteers. These data indicate that plant stanol supplementation in a healthy population does not modify immune responses. Further studies are required in larger populations with carefully selected volunteers to understand underlying mechanisms of plant stanol ester consumption on the immune system in HIV, asthma, and allergic patients that seem to be absent in immunologically healthy volunteers.
